# How the Pretreatment Temperature of Zeolitic Catalysts Can Affect the Reaction Temperature of Methanol to Olefins and Gasoline Processes

**DOI:** 10.3390/ma18061370

**Published:** 2025-03-20

**Authors:** Simón Yunes, Abel Gaspar Rosas, Antonio Gil

**Affiliations:** 1 Micromeritics Instrument Corporation, 4356 Communications Drive, Norcross, GA 30093, USA; abel.gaspar@micromeritics.com; 2 INAMAT^2-Departamento de Ciencias, Universidad Pública de Navarra, 31006 Pamplona, Spain; andoni@unavarra.es

**Keywords:** methanol, olefins, dehydration, temperature-programmed desorption (TPD), acidity

## Abstract

The dehydration of methanol to produce light olefins and gasoline, known as MTO (methanol-to-olefins) process requires acidic catalysts that maintain their acidity at reaction temperatures. Zeolites, such as SAPOs and ZSM-5, are commonly used for this purpose due to their acidic centers. The initial step in these experiments involves the activation or pretreatment of these solids to remove physically adsorbed water from their pores. Inadequate pretreatment can lead to the destruction of the existing Brönsted sites through the dihydroxylation of surface -OH groups. Therefore, it is crucial to pretreat the zeolites properly to preserve the Brönsted sites. One method is to subject the fresh catalyst to programmed dehydration, which involves desorption at a controlled temperature while monitoring the appearance of water that results from Brönsted site dihydroxylation. The temperature at which the dehydration peak appears determines the optimal reaction temperature. The results presented in this work will demonstrate the progressive deactivation of the catalysts when the reaction temperature exceeds 400 °C.

## 1. Introduction

The methanol-to-olefins (MTO) process has been recognized for many years as a significant method for converting methanol into more valuable products that are widely used in daily life, such as gasoline, aromatics, and other hydrocarbons. This process was first proposed by Mobil Corporation in the 1970s [1], marking a pivotal development in the field of chemical engineering. Since then, numerous companies have adopted and refined the MTO process [2,3,4,5]. Chang and Silvestri in 1977 developed a process similar to MTO and called it MTG or methanol-to-gasoline, in which compounds ranging from C_4_ to C_10_ are produced, which surpasses the Fischer–Tropsch process in the production of synthetic gasoline [6,7]. In the same way, Union Carbide in 1981 [8,9,10] made a great effort in these processes to respond to the global energy crisis of the 1970s, being the development of SAPO-based catalysts a key factor for the upscaling of MTO process.

In early 2010, the Dalian Institute of Chemical Physics of the Chinese Academy of Science made a groundbreaking achievement by constructing the world’s largest coal-to-olefins plant using a HSAPO-34-based catalyst [11,12]. This facility represents a major advancement in the utilization of coal and biomass materials to produce commercially valuable products. Researchers at the institute have extensively studied the reaction mechanisms involved in the MTO process and have successfully employed microporous and mesoporous acidic catalysts [13,14,15] to yield hydrocarbons and other products from coal and biomass, which are abundant in nature. In this process, methanol is initially dehydrated to form dimethyl ether (DME) and subsequently forms a mixture of olefins and aromatic compounds [16].

**Scheme 1:**
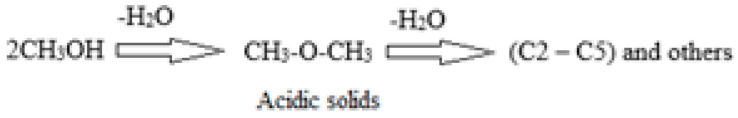
Dehydration of methanol in two steps as function of temperature to produce olefins and other products.

**Scheme 2:**
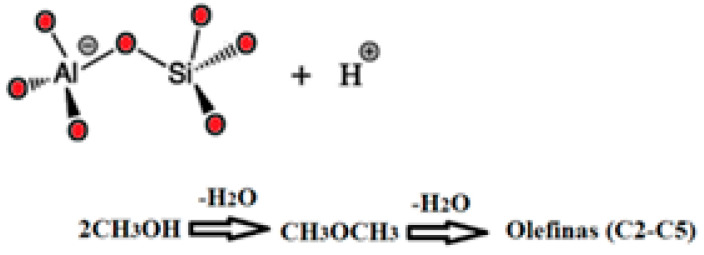
Possible mechanism of the MTO process where the H^+^ from the catalyst provokes the dehydration of the molecule of methanol.

The acidic catalysts used in the MTO process are typically zeolitic materials, known for their Lewis and Brönsted surface acid sites [17]. These sites play a crucial role in the reaction by facilitating the removal of a water molecule from methanol under the attack of the proton (H^+^) according to Scheme 1 and Scheme 2, resulting in the formation of dimethyl ether (DME) as the initial product [18,19,20,21]. As the reaction temperature continues to rise, a second water molecule is removed, leading to the production of olefins and other important molecules with greater added value as cycloparaffins and aromatics.

The Brönsted acid sites on the surface of the catalysts are -OH groups, which are highly sensitive to high temperatures. These sites can easily undergo dihydroxylation, producing water when the temperature exceeds certain thresholds. This dihydroxylation can lead to the loss of acidity and, consequently, catalytic activity. Therefore, it is essential to exercise caution during the pretreatment of these zeolitic solids before the reaction.

The pretreatment of zeolites typically involves heating them under a flow of inert gas to remove physically adsorbed water from their micropores. These micropores often contain atmospheric water and some hydrocarbons. The temperature used in this procedure is critical and must be carefully controlled to avoid exceeding the critical temperature that would cause dihydroxylation of the -OH groups. Proper pretreatment preserves the acidity and activity of the catalyst, which is vital for the efficiency of the MTO process.

Our main goal in this paper is to caution researchers to carefully select the degassing procedure for these materials prior to the reaction. Ensuring the correct pretreatment method is crucial to maintaining the integrity and effectiveness of the catalysts used in the MTO process. In our investigation, zeolite 3020, the most acidic, was degassed up to 250 °C and maintained at this temperature for 6 h. The dehydration signal was monitored by mass spectroscopy until the baseline returned to its original position. Under these conditions, the Brønsted acidity is expected to be affected, and consequently, the activity of this zeolite will also be impacted, as will be discussed later in this article. This sample will be denominated as 3020*, as it will be considered as an example for the conclusion in our research.

## 2. Experimental Procedure

Throughout this study, three zeolites from Zeolyst were selected, each with a distinct Si/Al ratio: 280 (CBV 28014), 80 (CBV 8014), and 30 (CBV 3020). These zeolites exhibit varying levels of acidity based on their Si/Al ratios, with a lower ratio indicating higher acidity. The physical gas adsorption technique was performed using a Micromeritics 3Flex Nocross, GA, USA) high vacuum system to characterize the texture of these zeolites. Nitrogen (Airgas, 99.999%) was used as the probe molecule for adsorption at −196 °C (liquid nitrogen temperature). This method provides detailed information about the surface area, pore volume, and pore size distribution of the zeolites, which are critical parameters affecting their catalytic performance [22].

**Figure 1:**
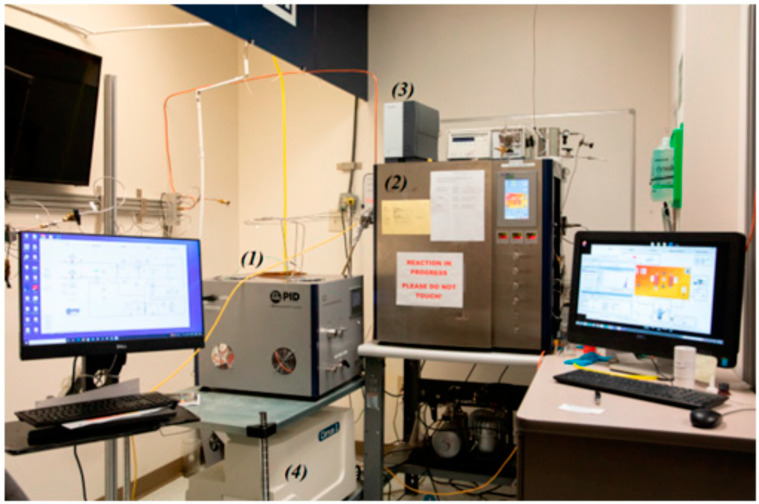
The equipment used in characterizing and testing the catalysts for this work: (1) the in situ catalyst characterization system (ICCS), (2) the FR-100 microreactor, (3) the micro-gas chromatograph, (4) the MKS mass spectrometer.

The rest of the characterizations and analyses, as well as the catalytic test, were carried out using an “in situ catalyst characterization system” (ICCS) that was connected to the inlet of the FR-100 reactor from PID-Micromeritics (PID & Eng, Madrid, Spain) (see Figure 1) [23]. This option provides comprehensive insights into the catalyst’s properties and behavior under reaction conditions, ensuring that the catalyst is well-understood and optimized for its intended application. A MKS Cirrus II mass spectrometer was connected to the reactor exhaust to monitor all the gases released. More information about these systems and their use in the characterizations and applications of the catalysts has been previously reported [24].

In order to determine the optimal reaction temperature without altering the Brönsted acid centers, water DTP (temperature-programmed desorption) experiments have been performed. This test is crucial for limiting the reaction temperature to prevent catalyst deactivation due to the loss of acidity. That is, a stream of 100 cm^3^/min of He was passed through the reactor containing the solid (0.4 g). The temperature of the material was then increased using a ramp of 10 °C/min from room temperature to 600 °C, in order to observe the dehydration of the zeolite.

The MTO reaction was performed using 0.3 g of the sample placed in the FR-100 reactor, connected to the mass spectrometer (MKS-Cirrus II (Andover, MA, USA)) to monitor the reaction effluent. For this study, a few olefins were considered to avoid complexity of the analysis. The following signals were then monitored: *m*/*z* 4 for helium, *m*/*z* 18 for water, *m*/*z* 28 for ethylene, *m*/*z* 31 for methanol, *m*/*z* 41 for propylene, and *m*/*z* 45 for dimethyl ether. The sample was first degassed at 120 °C under a flow of 100 cm^3^/min of He (Airgas, 99.999%) for 2 h before the reaction. Methanol (99.8%, Sigma-Aldrich) was pumped using an HPLC pump (Gilson) at a rate of 0.03 cm^3^/min through a gas mixer set at 120 °C and carried by He through the catalyst bed. The reactor pressure was set to 50 bars. This combination of methanol flow and catalyst mass yielded a space velocity (HGSV) of about 4 h^−1^. Upon establishing a stable baseline for methanol on the mass spectrometer, the reactor temperature was ramped up to 350 °C initially and then to 500 °C at a rate of 3 °C/min. As the catalyst bed temperature increased, the reaction products began to form and were detected by the mass spectrometer. This setup allowed for precise control and monitoring of the reaction conditions, ensuring accurate and reproducible results. The performance of the catalyst was determined by the difference between the initial partial pressure of methanol measured by the mass spectrometer and the final signal. This measurement provided a quantitative assessment of the catalyst’s ability to convert methanol into the desired products.

## 3. Experimental Results

**Figure 2:**
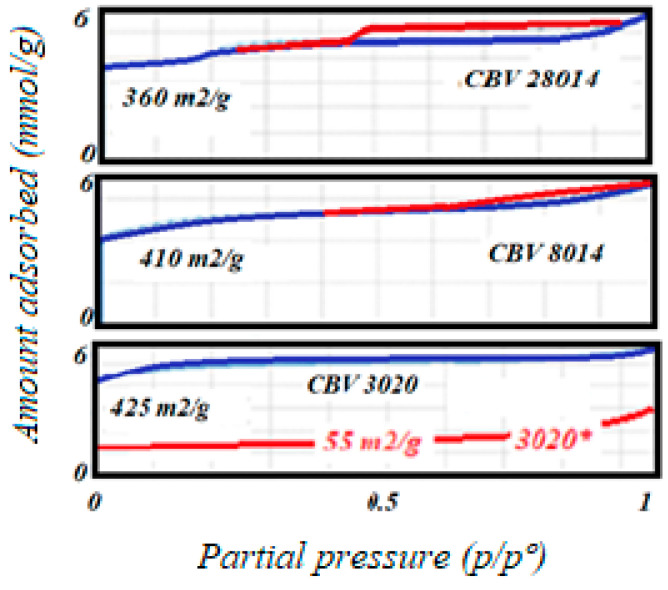
N_2_ adsorption–desorption at −196 °C of the zeolites studied.

The gas adsorption technique results indicate that all studied solids exhibited type I adsorption isotherms (see Figure 2). This type of isotherm is characteristic of microporous materials, such as zeolites, which have pore sizes less than 2 nm in diameter. Type I isotherms typically show a steep initial uptake of gas at low relative pressures, reflecting the filling of micropores [25]. The zeolite with a higher Si/Al ratio (CBV28014) displayed a small hysteresis loop in its adsorption isotherm. This suggests the presence of some mesoporosity, likely due to the higher silica content in the sample. Mesopores are larger than micropores, typically ranging from 2 nm to 50 nm in diameter, and their presence can enhance the material’s overall surface area and accessibility.

Zeolites are known for their highly microporous structure, which results in a high surface area and significant microporous volumes. These properties are summarized in Table 1, which lists the surface area and pore volume measurements for each zeolite.

**Figure 3:**
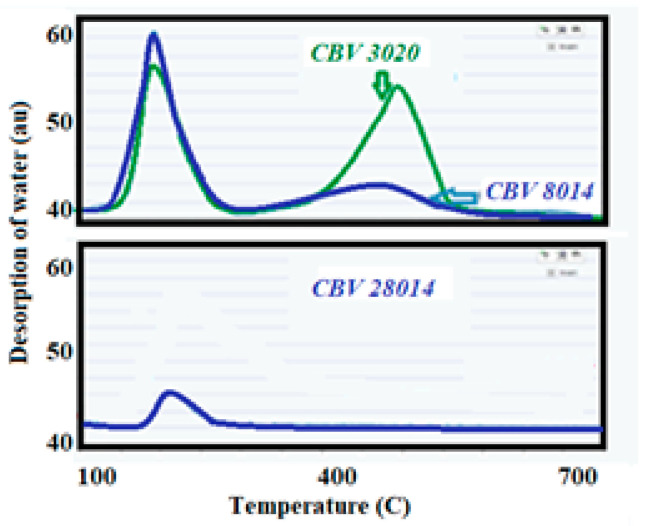
Temperature desorption profiles of water.

The DTP results obtained are shown in Figure 3, where two water desorption maxima can be observed, each corresponding to different processes occurring within the zeolitic materials. The experimental procedure carried out has been described in the Experimental Section. The first peak that appears, 140 °C, is attributed to the physically adsorbed water. The large micropore volume of the zeolitic materials facilitates the adsorption of water molecules, which are then released at this temperature. This process indicates the presence of a substantial amount of physically adsorbed water within the micropores. The second-highest temperature peak, 415 °C, corresponds to the dehydroxylation of -OH groups from the surface of the zeolitic materials. The temperature just before this second peak is considered the optimal reaction temperature for the MTO process. At this temperature, the zeolitic materials maintain their acidity, which is crucial for catalytic activity. However, beyond this temperature, the samples are likely to lose their acidity due to the destruction of the Bronsted acid sites. This loss of acidity can lead to the deactivation of the catalyst, even before the reaction occurs, thereby affecting the efficiency of the MTO process. However, the zeolite sample pre-treated at 300 °C for 4 h exhibited a markedly different spectrum compared to previous samples (experimental results found not shown). The first peak appeared at a temperature similar to that of the other studied samples. Furthermore, the desorption profile did not remain constant but gradually diminished over time. This gradual fading continued until the profile eventually returned to the original signal, indicating a complete desorption process. This behavior is clearly illustrated in Figure 3, where the changes in the spectrum can be observed in detail. Understanding these desorption peaks is essential for optimizing reaction conditions and ensuring the longevity and effectiveness of the catalyst in the MTO process. The desorption signal was monitored by a mass spectrometer connected to the reactor exhaust. The mass spectrometer tracked the signal of *m*/*z* = 18, which is typical of water. This monitoring helps in understanding the dehydration behavior and the stability of the catalyst under different temperature conditions.

**Figure 4:**
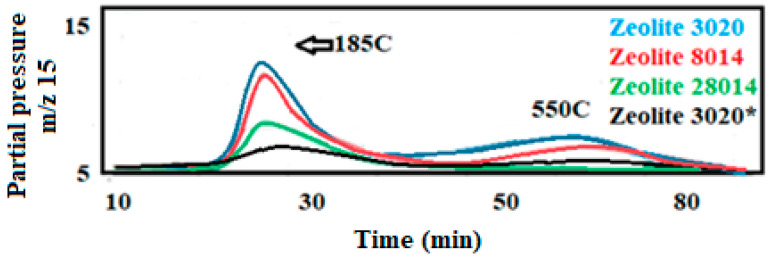
Temperature desorption profiles of NH_3_.

The acidity of the solids was assessed by the amount of ammonia desorbed in a temperature-programmed ramp (NH_3_-TPD) in He [23]. The zeolites were carefully pre-treated at 350 °C (temperature below the appearance of the second dehydration peak) for 3 h using a stream of He. The temperature was then lowered to 75 °C, and a stream of He with 10% in NH_3_ was passed through for 30 min. This was considered to be sufficient time to ensure complete saturation of the solid. The surface of the zeolite was then cleaned by a stream of He at a flow rate of 100 cm^3^/min, the objective of this cleaning being to completely eliminate the physically adsorbed NH_3_. From here, the reactor containing the sample begins to be heated with a ramp of 10 °C/min until reaching a temperature of 600 °C where a complete spectrum of ammonia desorption is obtained (see Figure 4). The desorbed ammonia was monitored by the mass spectrometer using the *m*/*z* 15 signal to avoid confusion with *m*/*z* 17 of water. This method provides detailed information about the strength and distribution of acidic sites on the catalyst surface. The TPD profiles revealed significant differences in acidity, which are directly related to the Si/Al ratio of the samples. Samples with low Si content (Si/Al ratios of 80 and 30, CBV 8014 and CBV 3020) exhibited two acid sites. The first maximum, which appears around 185 °C, can be related to weak acid centers, while the second maximum appears around 405 °C and could correspond to strong acid centers. Samples with high Si content (Si/Al ratio of 280, CBV 28014) exhibited an acid site at low temperature, indicating moderate acidity. The presence of a single peak suggests a more uniform distribution of acid sites, which are moderately strengthened. However, this moderate acidity does not significantly contribute to the ongoing reaction, which occurs at about 350 °C. This implies that while the sample has some acidic properties, they are not sufficient to drive the reaction effectively at the required temperature. The differences in the TPD profiles between samples with low and high Si/Al ratios underscore the impact of silicon content on the acidity and catalytic behavior of the samples. Understanding these differences is essential for optimizing the catalytic performance for specific reactions. If the amounts of NH_3_ desorbed are compared to the areas under the curves, very different values are observed between the three zeolites with a factor of almost 20 times higher for zeolite CBV 8020 (see Table 1). These differences can be related to the Si/Al content in each of the zeolites, lower Si content, and higher acidity. The results suggest that zeolites with a Si/Al ratio lower than 280 are more suitable for the MTO reaction. This is because the acid sites in these zeolites are crucial for the dehydration of methanol, leading to the production of olefins and other aromatics. In conclusion, this study of acidity highlights the importance of the Si/Al ratio in determining the acidity of zeolites. Only those with a Si/Al ratio below 280 possess the necessary acid sites to effectively catalyze the MTO reaction, thereby optimizing the production of desired hydrocarbons.

**Figure 5:**
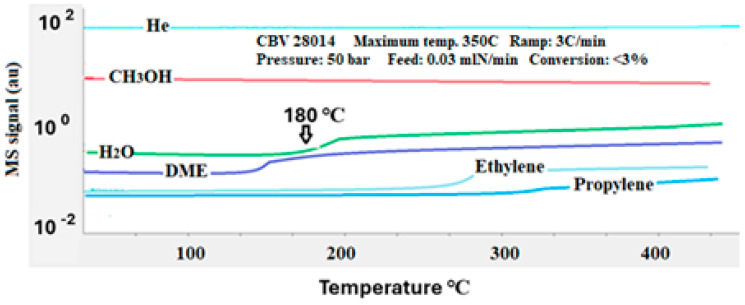
MS signals of the different effluents for the zeolite CBV 28014 (Si/Al = 280).

The performance of the catalyst with the highest Si/Al ratio of 280 (CBV 28014), which also has the weaker acidity, was included in Figure 5. This catalyst successfully produced DME (dimethyl ether) at around 200 °C. However, only trace amounts of olefins were detected near 350 °C, which is considered the optimal reaction temperature based on the acidity profiles shown above in Figure 4. From this result, it is possible to conclude that zeolites with a high Si/Al ratio are not effective for producing olefins from methanol, as the conversion rate was less than 3%. This suggests that the weaker acidity of these catalysts is insufficient to drive the further conversion of DME to olefins.

**Figure 6:**
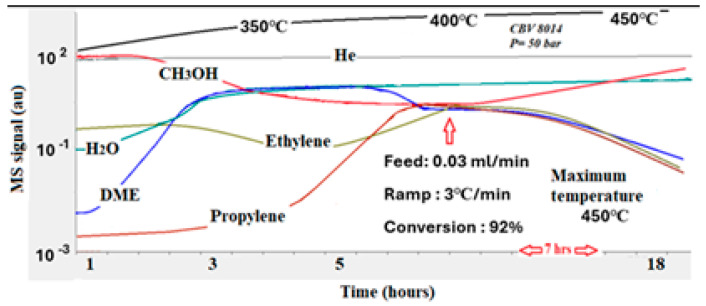
MS signals of the different effluents for the zeolite CBV 8014 (Si/Al = 80).

**Figure 7:**
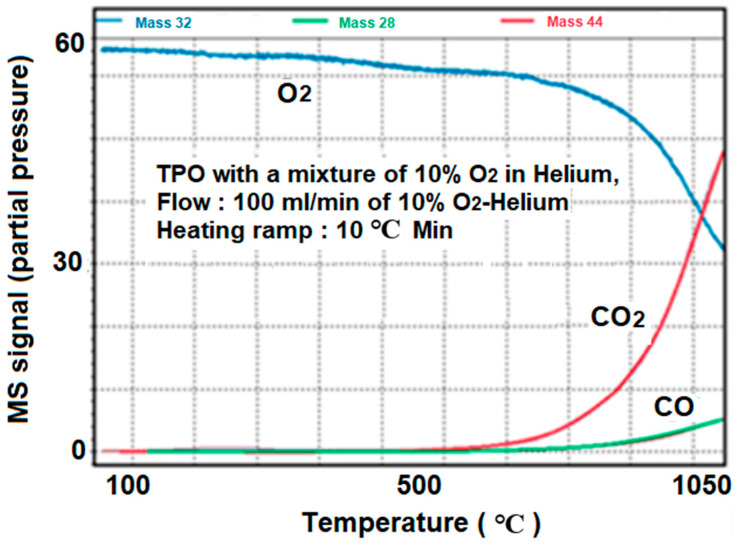
MS signals corresponding to the TPO results in producing CO and CO_2_ as carbon being oxidized by the O_2_ mixture.

**Figure 8:**
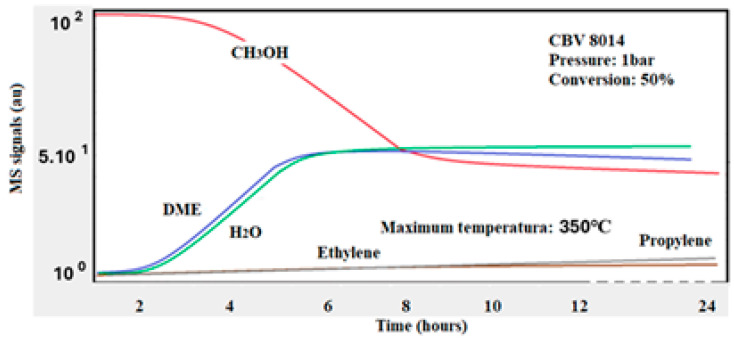
MS signals of the different effluents for the zeolite CBV 8014 (Si/Al = 80) at 1 bar of pressure.

The performance of the zeolite with a Si/Al ratio of 80 (CBV 8014) carried out at a pressure of 50 bar was presented in Figure 6. This sample exhibited moderate acidity, as shown in Table 1. Similarly to the previous catalyst, it produced DME at around 200 °C. As the temperature approached 350 °C, both ethylene and propylene were produced, with a methanol conversion rate of nearly 92%. This high conversion rate indicates that moderate acidity is more effective in facilitating the MTO process. However, as the temperature exceeded 400 °C, all catalysts began to deactivate. This deactivation was indicated by the methanol signal on the mass spectrometer returning to its original level. The deactivation can be due to two main phenomena: (a) the possible formation of large hydrocarbon molecules that block the pores where the acid sites are located, thus minimizing contact between reactant molecules and active sites, and (b) the formation of graphite, which covers the catalyst surface and hinders the active sites, leading to a rapid deactivation, as will be demonstrated in Figure 7. In this experiment, the oxygen is consumed at high temperatures. As result, carbon dioxide and carbon monoxide were detected. The incomplete oxidation process should be attributed to the fact that graphite requires significantly high temperatures to be fully oxidized. The performance of the same catalyst (Si/Al = 80) at atmospheric pressure was summarized in Figure 8. In this scenario, the catalyst converted almost 50% of methanol, producing only DME, with no olefins formed. This indicates that the MTO process requires high pressure to effectively convert methanol into olefins. The absence of graphite formation, as no CO_2_ or CO was detected under the effect of O_2_ by TPO (temperature-programmed oxidation), suggests that at low pressure, the catalyst deactivates less and maintained its activity for almost 24 h but is not suitable for the MTO process to produce olefins.

Deactivation of the catalyst was indicated when the methanol signal returned to its initial partial pressure. Upon deactivation, the catalyst bed at 500 °C was swept with a flow of 100 cm^3^/min of He to remove any remaining traces of methanol or other products. The reactor temperature was then lowered to 100 °C to proceed with temperature-programmed oxidation (TPO). This technique was used to investigate the presence of carbonaceous materials produced during the reaction, which could cover the catalyst surface and cause deactivation. A flow of 100 cm^3^/min of 5% O_2_ in He was used to flow over the catalyst bed, and the temperature was raised to 1050 °C. Partial oxidation of the carbonaceous materials was detected by the mass spectrometer as CO_2_ and CO, limited by the oven’s ability to reach higher temperatures required to oxidize graphite. This step ensured that any potential deactivation due to carbon deposition was thoroughly investigated and addressed.

**Figure 9:**
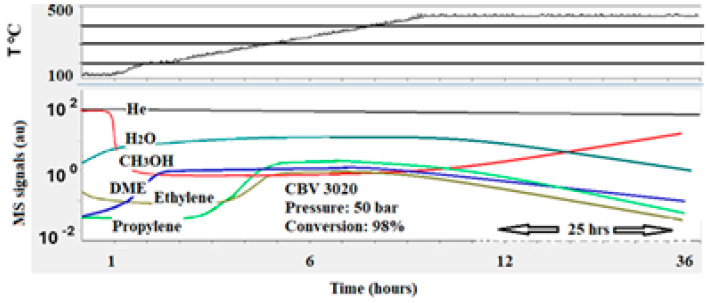
MS signals of the various effluents for the zeolite CBV 3020 (Si/Al = 20).

The performance of the most acidic catalyst (Si/Al = 30), which converted nearly 100% of methanol to olefins and other products, was included in Figure 9. As observed previously, DME was the first product formed at nearly 200 °C. As the temperature approached 350 °C, both ethylene and propylene were produced, with conversion reaching 100%. However, this highly active catalyst had a shorter lifespan compared to the moderately acidic catalyst. This is likely due to the faster formation of larger hydrocarbon molecules and graphite, which are detrimental to the catalyst’s activity. The rapid deactivation of this catalyst underscores the trade-off between high activity and stability in the MTO process.

**Figure 10:**
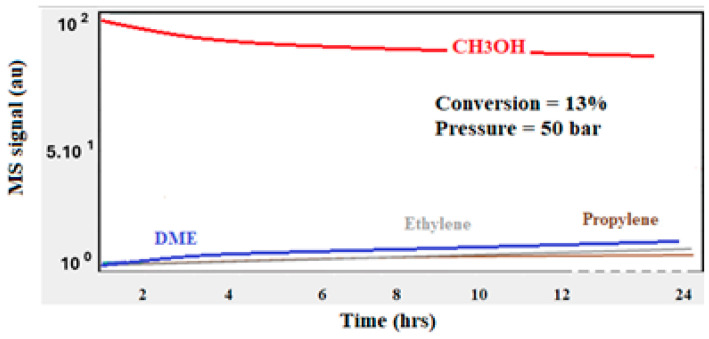
MS signals of the different effluents for the zeolite CBV 3020*.

The performance of zeolite CBV 3020* after undergoing a treatment at 250 °C for 6 h was summarized in Figure 10. This specific treatment was designed to reduce the strong acidity of the sample. The purpose was to investigate the effects of an uncontrolled degassing temperature on the sample’s activity during the reaction process. The treated catalyst did not produce the expected olefins. Instead, the reaction only advanced through the initial dehydration step. This step resulted in the formation of a dimethyl ether (DME) at a temperature of 350 °C. The absence of olefin production suggests that the treatment conditions, particularly the degassing temperature, significantly affected the catalytic performance of the zeolite. This observation highlights the critical role of precise temperature control in the degassing process. Uncontrolled temperatures can lead to suboptimal catalytic performance, as evidenced by the limited reaction pathway observed in this experiment. Further studies are needed to optimize the treatment conditions to achieve the desired catalytic outcomes.

## 4. Conclusions

In conclusion, the methanol-to-olefins (MTO) process is highly dependent on the acidity and Si/Al ratio of zeolite catalysts. Catalysts with a high Si/Al ratio and weaker acidity tend to be less effective in producing olefins, which are crucial intermediates in the chemical industry. In contrast, catalysts with moderate acidity and an appropriate Si/Al ratio exhibit higher conversion rates and better performance, especially under high-pressure conditions. This is because the optimal balance of acidity and Si/Al ratio facilitates the desired chemical reactions.

One of the primary challenges in the MTO process is the formation of graphite and large hydrocarbons, which can block the active sites of the catalyst and reduce its effectiveness. These by-products highlight the need for careful optimization of reaction conditions, such as temperature and pressure, to maintain catalyst performance and longevity. By understanding and controlling these factors, it is possible to extend the life of the catalyst and improve the overall efficiency of the MTO process.

The findings from these experiments provide valuable insights into the design and optimization of catalysts for the MTO process. By balancing the acidity and Si/Al ratio, and optimizing reaction conditions, such as pressure and temperature, it is possible to enhance the efficiency and stability of the MTO process. This paves the way for more sustainable and efficient production of olefins from methanol, which is a key step towards reducing reliance on fossil fuels and lowering the environmental impact of chemical manufacturing.

Additionally, special caution must be taken when degassing or activating the catalyst at specific temperatures. Highly microporous materials, such as zeolites and SAPOs (silicoaluminophosphates), often require extended degassing times to ensure the removal of physisorbed water from their micropores. However, it is crucial to maintain a low temperature during this process, not exceeding 120 °C. Using a higher temperature to speed up the desorption of physisorbed water can lead to the destruction of the Brønsted acid sites within the catalyst. These sites are essential for the catalyst’s activity, and their destruction would render the catalyst ineffective before the reaction even begins. Therefore, careful temperature control is necessary to preserve the integrity and functionality of the catalyst throughout the MTO process.

In summary, the successful implementation of the MTO process hinges on the careful selection and optimization of catalyst properties and reaction conditions. By addressing the challenges associated with catalyst deactivation and ensuring proper activation procedures, it is possible to achieve a more efficient and sustainable production of olefins, contributing to the advancement of green chemistry and industrial sustainability.

## Figures and Tables

**Table 1:** Textural properties derived from N_2_ adsorption at −196 °C and NH_3_ desorbed.

Catalyst	Si/Al Ratio	S_BET_ (m^2^/g)	V_p_ (cm^3^/g) ^a^	NH_3_ (mmol(STP)/g)
CBV 3020	30	425	0.173	0.83
CBV 8014	80	410	0.154	0.48
CBV 28014	280	360	0.193	0.04
CBV 3020*		55	0.0104	0.13

^a^ Total pore volume, calculated from Ar adsorption at p/p^o^ = 0.98.

## Data Availability

All the data are available within the manuscript.
